# A “bullous” syncope: When the air is too much

**DOI:** 10.1016/j.hrcr.2024.07.015

**Published:** 2024-07-18

**Authors:** Raimondo Calvanese, Claudio Capobianco, Carmen D’amore, Gennaro Izzo, Michelangelo Canciello, Mario Di Stasio, Bernardino Tuccillo

**Affiliations:** ∗ Department of Cardiology, Ospedale del Mare, Asl Napoli 1 centro, Naples, Italy; † Department of Cardiology, Ospedale Santa Maria delle Grazie di Pozzuoli, Naples, Italy; ‡ Department of Thoracic Surgery, Ospedale del Mare, Asl Napoli 1 centro, Naples, Italy

**Keywords:** Syncope, Vanishing lung syndrome, Giant pulmonary bulla, Asystolic pause, Emphysema

**Key Teaching Points**

• In most cases of syncope the identification of etiology is made with noninvasive diagnostic tools, but it can be extremely challenging for the remaining cases.
• In rare cases the syncope can be caused by the stimulation of the left vagus nerve caused by extrinsic compression along its course.
• The giant bulla may be a rare cause of reflex syncope, and such a condition should be searched for in difficult cases.

## Introduction

The most frequent type of syncope in young adults is reflex or neurally mediated syncope, related to an anomalous balance between the sympathetic and parasympathetic nervous system, which is responsible for a temporary cerebral hypoperfusion.

The identification of etiology is found in approximately two-thirds of these cases with noninvasive diagnostic tools. For the remaining cases the diagnosis can be extremely challenging and can necessitate more in-depth investigations.

## Case report

**Figure 1:**
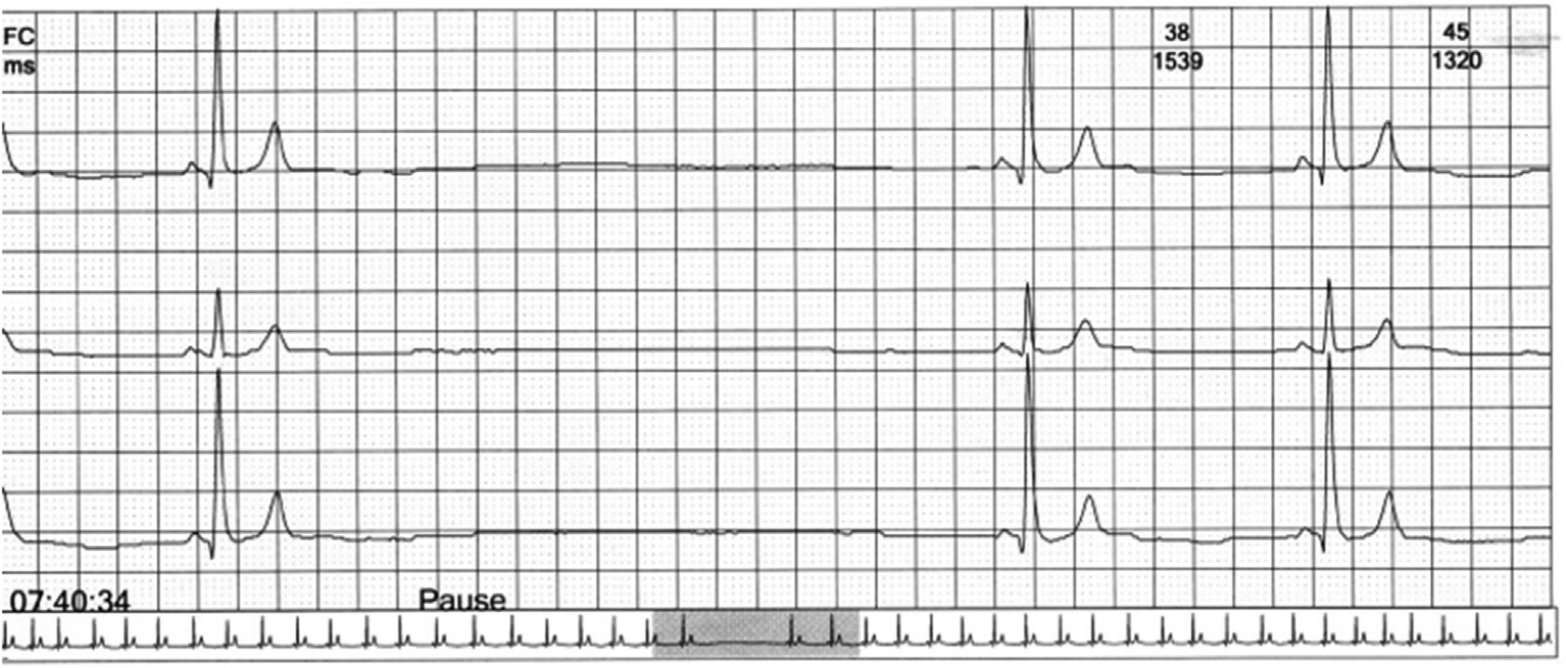
The electrocardiogram highlights a 4-second pause in the sinus rhythm, which is accompanied by chest pain and a feeling of faintness.

We report the case of a 38-year-old man who presented to the emergency department complaining of syncope preceded by chest and epigastric pain without fever or dyspnea. His past medical history revealed, for about a year, 3 syncopal episodes, at rest and while standing, sometimes following an intense epigastric pain, as well as various episodes of presyncope. The patient was an active smoker (about 20 packs/year), without other cardiovascular risk factors. The Holter monitoring performed before our observation showed recurrent daytime asystolic pauses of up to 4 seconds (Figure 1).

The initial evaluation at the emergency department, which comprised physical examination, electrocardiography, echocardiography, and laboratory testing, including high-sensitivity troponin, did not yield any pathological finding and presented stable hemodynamic parameters.

**Figure 2:**
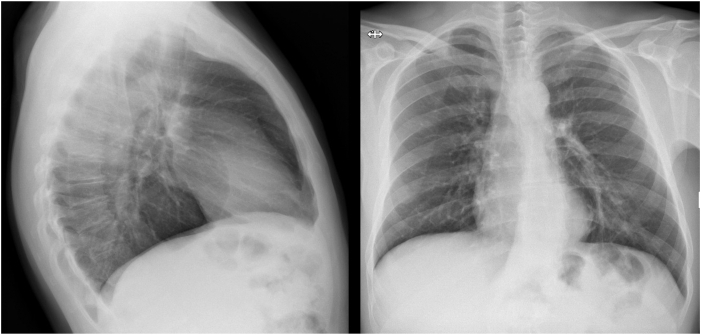
Left-lateral (top) and posteroanterior (bottom) chest radiographs show a bullous lesion in left-posterior mediastinal region.

**Figure 3:**
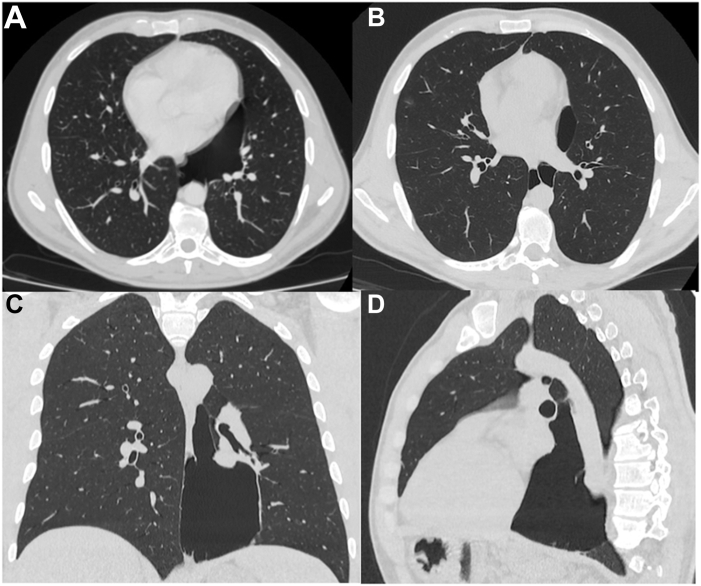
Transversal (**A,B**), coronal (**C**), and lateral (**D**) sections of high-resolution chest tomography highlight a wide air bulla in left-posterior mediastinal region in direct relationship with the pericardium and esophagus medially and anteriorly and with the diaphragm inferiorly.

The lateral view of the chest radiography revealed a giant bulla in the posterior mediastinum (Figure 2). Owing to this finding, the patient was admitted to the cardiology department. High-resolution chest tomography confirmed the presence of a giant bullous area, localized between the posterior mediastinum and the inferior left lobe, strictly related to the pericardium and esophagus anteriorly and medially, and vanishing lung syndrome (VLS) was diagnosed (Figure 3).

The diagnostic work-up of epigastric pain by esophagogastroduodenoscopy and abdominal ultrasound were completely normal.

The head-up tilt test, performed to exclude a vasovagal syncope, was negative.

In an interdisciplinary discussion made up of cardiologists, radiologists, and thoracic surgeons, a thoracoscopic bullectomy was proposed to the patient despite the lack of data reported in scientific literature.

A left videothoracoscopic bullectomy (Supplemental Figure 1) was performed without complication and the patient was discharged 7 days later. Repeated 24-hour Holter monitoring did not evidence any recurrence of bradyarrhythmia. A 2-year follow-up did not show any recurrence of faintness or syncope, and the patient no longer suffered from epigastric pain.

## Discussion

Syncope is one of the most common reasons for emergency department clinic visits.^1^

The differential diagnosis of recurrent syncope can range from its most common benign cause, neural/reflex, to life-threatening cardiac arrhythmias. Identification of etiology is found with noninvasive diagnostic tools in approximately two-thirds of these cases but can be extremely challenging for the remaining group.

VLS is a chronic condition defined by a giant emphysema in the thorax cavity, occupying at least one-third of the hemithorax and compressing the surrounding normal lung parenchyma, with subsequent mediastinal shift.^2^^,^^3^

The main etiological factors include male sex, cigarette and marijuana smoking, alpha-1-antitrypsin deficiency, and connective tissue diseases such as Marfan and Ehlers-Danlos syndromes.

VLS is associated with a wide spectrum of clinical manifestations, including worsening dyspnea, cough, declining pulmonary function, and occasionally spontaneous pneumothorax owing to rupture of bullae.^2^^,^^3^

The treatment is conservative in asymptomatic patients and surgical in patients with worsening pulmonary function, increased size of air bullae, pneumothorax, recurrent infection, or lung hemorrhage. However, differentiating a pneumothorax from VLS is very important because the management differs, in the case of VLS, avoiding chest tube placement and iatrogenic pneumothorax. In general, patients should be counseled to stop smoking tobacco.^4^^,^^5^

The etiological factors identified in our patient were male sex and active smoking. The alpha-1-antitrypsin deficiency test was negative, and he had neither the typical clinical signs nor echocardiographic, osteoarticular and ocular signs of Marfan syndrome. The clinical manifestations reported by the patient were atypical and not associated with respiratory failure.

Syncope mediated by vagal nerve activation owing to mechanical compression by a head or neck mass is not uncommon, but compression by a mediastinal mass is rare.

Theoretically, any abnormal structure in close proximity to the left atrial wall could cause a direct compression of the left atrium, but the poor filling of the right ventricle by extrinsic mass is uncommon. The 4 abnormal structures that could compress the left atrium are gastrointestinal, mediastinal, intracardial/pericardial and pulmonary structures.^6^

DeLuca and colleagues^7^ reported the extrinsic compression of the left atrium by a lung tumor and secondary impaired left atrial filling, leading to pulmonary edema.

A huge hiatal hernia that caused compression of the left atrium and syncope has also been reported.^8^ Also, a rapid increase of intrathoracic pressure by a spontaneous pneumothorax may cause abnormal parasympathetic nervous system activation and atrioventricular block.^9^

Similarly to our case, Massaro and colleagues^10^ reported a case of complete atrioventricular block in a healthy young woman identified as continuous stimulation of the left vagus nerve caused by extrinsic compression by thymic hyperplasia.

Some cases of lung cancer have been associated with syncope and bradycardia; pathophysiologic hypotheses include compression of the left vagus nerve or its cardiac branches.^11^^,^^12^

The association between chest bulla and asystole owing to reflex syncope has not previously been described.

In this case, the giant bulla has an atypical location, as it is usually described in a more peripheral chest position. In our case, probably the syncope was due to a synergistic effect of different pathophysiologic mechanisms.

We think that the giant bulla can cause syncope through a reflex mechanism triggered either by direct vagal nerve branch stretch or by abdominal pain. Unfortunately, it is difficult distinguish between the 2 components and attribute the right amount to each one. The atypical location of the bulla could certainly play a role.

During the thoracoscopic bullectomy (Supplemental Figure 1) the close relationship between the giant bulla and the left vagus nerve has been highlighted. Once the bulla had been sectioned it was possible to see the left vagus nerve inside the bulla. It is likely that the pressure of the bulla on the left vagus nerve can cause an increase in afferent neural activity, especially afferent vagal nerve (C-fiber) and mechanoreceptor activity, and produce a centrally mediated paradoxical decrease in heart rate and peripheral vasodilatation, resulting in neurally mediated syncope.^6^^,^^7^

Furthermore, its proximity with the diaphragm could cause epigastric pain and could intensify an abnormal dysautonomic response and secondary alterations of the heart rhythm.

Although the association between giant thoracic bullae and lipothymic episodes is described in the literature, to date this is the first case in which thoracic bulla causes syncope owing to sinus node arrests.

## Conclusion

Syncope is a recurring symptom in the young population and a neuromediated origin is highly frequent. In most cases the identification of etiology is found with noninvasive diagnostic tools, but it can be extremely challenging for the remaining cases. A negative initial work-up deserves further evaluation. We describe the rare association between a giant bulla, with an atypical location in posterior mediastinum, and syncopal episodes from asystolic pauses, successfully treated by thoracoscopic bullectomy.

## Disclosures

The authors have no conflicts to disclose.

## Appendix Supplementary Data

Supplemental Figure 1 A: Thoracoscopic view of the Giant Bulla: the red star shows the epicardial surface of the left ventricle; the white star shows the Clamp that lifts the wall of the opened bulla; the green star shows the left lower lobe lung. **B:** Thoracoscopic view: the black lines delimit the border of the opened Giant Bulla; the white star shows the close relationship between the Bulla and the left vagal nerve (the left vagal nerve is inside the Giant Bulla); the dashed white line shows the vagal nerve course;
