# Supplementary material for: A “bullous” syncope: When the air is too much
Source: HeartRhythm Case Rep. 2024 Jul 18;10(10):748–51. doi: 10.1016/j.hrcr.2024.07.015 (PMC11628835; doi:10.1016/j.hrcr.2024.07.015)
Supplement: Supplemental Figure 1 — A: Thoracoscopic view of the Giant Bulla: the red star shows the epicardial surface of the left ventricle; the white star shows the Clamp that lifts the wall of the opened bulla; the green star shows the left lower lobe lung. B: Thoracoscopic view: the black lines delimit the border of the opened Giant Bulla; the white star shows the close relationship between the Bulla and the left vagal nerve (the left vagal nerve is inside the Giant Bulla); the dashed white line shows the vagal nerve course; [file mmc1.docx]

**A B**

**Fig.S1 A:** Thoracoscopic view of the Giant Bulla:

the red star shows the epicardial surface of the left ventricle;

the white star shows the Clamp that lifts the wall of the opened bulla;

the green star shows the left lower lobe lung.

**Fig S1 B:** Thoracoscopic view:

the black lines delimit the border of the opened Giant Bulla;

the white star shows the close relationship between the Bulla and the left vagal nerve (the left vagal nerve is inside the Giant Bulla);

the dashed white line shows the vagal nerve course;
